# Choroidal thickness changes in patients with systemic lupus erythematosus treated with hydroxychloroquine using three dimensional maps

**DOI:** 10.1007/s10792-026-03974-3

**Published:** 2026-02-09

**Authors:** Francisco de Asís Bartol-Puyal, María Chacón González, Damián García Navarro, Borja Arias-Peso, Silvia Méndez-Martínez, Luis Pablo

**Affiliations:** 1 Universidad de Zaragoza, Calle Pedro Cerbuna, 12, 50009 Zaragoza, Spain; 2 Miguel Servet University Hospital, Paseo Isabel la Católica, 1-3, 50009 Zaragoza, Spain; 3 University of Zaragoza, Zarogoza, Spain; 4 Biotech Vision SLP (Spin-Off Company), University of Zaragoza, Zaragoza, Spain

**Keywords:** Choroidal thickness, Hydroxychloroquine, Swept-source optical coherence tomography, Systemic lupus erythematosus

## Abstract

**Purpose:**

To compare variations in choroidal thickness (CT) between patients with systemic lupus erythematosus (SLE) treated with hydroxychloroquine (HCQ) and healthy subjects for at least one year.

**Methods:**

Cross-sectional study enrolling patients between 30 and 55 years of age with SLE and treated with HCQ for at least one year, and aged-matched healthy subjects. Exclusion criteria included any other ophthalmological disorder or previous treatment, and systemic treatment with immunosuppressive drugs or a dose of corticosteroids higher than 5 mg/d. Macular CT was measured automatically in a grid of 30 × 30 cubes using Triton swept-source optical coherence tomography (Topcon). These were merged into 25 zones (each one comprising 6 × 6 cubes). Three-dimensional (3D) CT maps were created using Microsoft Excel and mean CT values in the grid of 30 × 30 cubes. One eye of each patient was randomly selected for the study.

**Results:**

60 patients and 54 healthy subjects were recruited. Mean age was 45.16 ± 6.43 and 43.79 ± 8.98 years (*p* = 0.346), respectively. Mean axial length was 23.52 ± 0.96 and 23.67 ± 0.87 mm (*p* = 0.137), respectively. Mean SLE duration was 125.58 ± 63.10 months, and mean duration of HCQ was 87.87 ± 52.13 months. There were no differences in intraocular pressure (*p* = 0.271) or spherical equivalent (*p* = 0.219). Choroidal zones number 9, 14, 15, 19 and 20 (central nasal locations) were thicker in SLE patients. Neither SLE duration, nor HCQ duration had any influence on CT (*p* > 0.05).

**Conclusions:**

Patients with SLE treated with HCQ for one or more years present higher CT values than healthy subjects. Nasal zones seem to be the most sensitive to these changes, while the other choroidal locations remain similar apparently. However, neither duration of SLE nor duration or dose of HCQ treatment seemed to have any influence on CT.

## Introduction

Systemic lupus erythematosus (SLE) is a disease that may affect the musculoskeletal system, the skin, the nervous system, the cardiovascular system, lungs, kidneys, and eyes, among others. It usually starts in young women, and its prevalence is estimated between 1.5 and 7.4 cases per 100,000 person-years [1]. Different autoantibodies are present in blood, such as anti-nuclear antibodies, anti-DNA (anti-deoxyribonucleic acid), anti-Sm (anti-Smith), anti-Ro, or anti-La. Hydroxychloroquine (HCQ) is usually a first-line treatment because it increases life expectancy [2, 3].

Although skin affection and dry eye disease are the most common, other ophthalmological disorders may occur. SLE can cause retinal microangiopathy, retinal vasculitis, or occlusion of retinal vessels [4]. A Purtscher-like retinopathy has also been described in these patients, as well as a coroidopathy [5], with implication of anti-retinal pigmented epithelium antibodies.

Hydroxychloroquine is well-known for its ophthalmological toxicity due to its high affinity towards melanin [6]. It usually starts in parafoveal locations, and finally extends to the entire macula. Once it appears, it might not stop despite discontinuing treatment with HCQ. Therefore, patients with SLE treated with HCQ can present retinal affection secondary to HCQ or to SLE itself.

Choroidal thickness (CT) may vary in patients with SLE without ophthalmological affection [7, 8]. Additionally, HCQ has a significant influence on CT [9], as well. In addition, CT may vary with age, multiple conditions, diseases and treatments. In most studies, CT is measured in one of the three following ways. First, manual measurements on horizontal optical coherence tomography (OCT) slabs. Second, automatic measurements on horizontal OCT slabs. Third, automatic measurements in every sector of the ETDRS (Early Treatment Diabetic Retinopathy Study) grid. Although they are adequate methods, the choroid may vary differently depending on the condition or disease, as it has been reported in case of age, diabetic retinopathy or high myopia [10]. Previous research showed that CT can vary differently depending on the location where it is measured [10–12], so the most adequate way to assess CT changes should be analyzing the entire macula. In case of SLE or HCQ, no detailed study has ever been conducted, as far as we know. Furthermore, no three-dimensional (3D) representations have ever been published.

The aim of this study is to compare variations in CT between patients with SLE treated with HCQ and healthy subjects for at least one year.

## Methods

A cross-sectional study was conducted after receiving approval from the regional ethics committee (EPA19/063). It adhered to the tenets of the Declaration of Helsinki. Inclusion criteria were Caucasian patients between 30 and 55 years of age diagnosed of SLE, and being treated with HCQ for one or more years. Age-matched healthy controls were enrolled afterwards. Exclusion criteria included amblyopia, any ophthalmological disorder or previous ophthalmological surgery, any systemic disease different from SLE, immunosuppressive drugs, systemic corticosteroids at a dose higher than 5 mg/d, pregnancy or puerperium. Patients diagnosed of active phase of SLE in the last year were excluded. Active phase was defined according to SLEDAI (Systemic Lupus Erythematosus Disease Activity Index) criteria and assessed by an experienced rheumatologist. One eye of each participant was randomly selected for the study. Evidence of retinal toxicity secondary to HCQ was an exclusion criterion if detected in funduscopy, OCT, visual field and/or electroretinogram. Sample size was calculated considering the following conditions: bilateral test, 95% level of confidence, statistical power of 90%, precision of 10 μm, variance of 500μm^2^. Therefore, sample size should be 105 patients.

Patients were examined at the same day time (between 16.00 and 20.00 h) by the same examiner between November 2020 and April 2022. They underwent a deep ophthalmological examination, and their medical records were revised to ensure they met inclusion criteria, but no exclusion criteria. Patients with SLE treated with HCQ were additionally examined with a visual field to discard possible retinopathy secondary to SLE or HCQ. The examination included mesopic best corrected visual acuity (BCVA) with ETDRS charts at a four-meter distance (logMAR scale), intraocular pressure (IOP) with Goldmann tonometry, refraction with refractometer, and axial length (AL) measurement with IOLmaster 500 (Carl Zeiss, Jena, Germany).

**Fig. 1:**
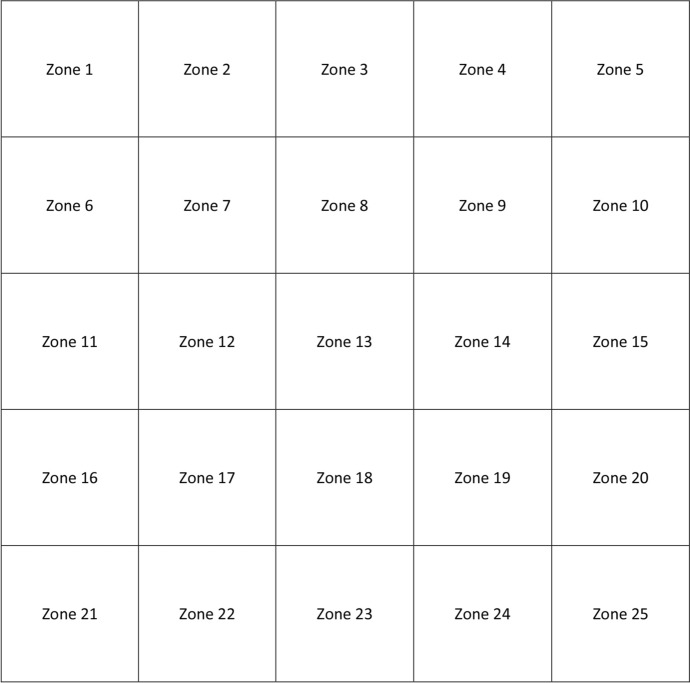
Choroidal division of the macula in a right eye model

Finally, they were examined with Triton DRI swept-source OCT version 1.1.7 (Topcon Corporation, Tokyo, Japan). A 7 × 7 mm fovea-centered macular cube analysis was performed, and internal software measured CT automatically. These CT measurements were obtained in a grid of 30 × 30 small cubes, which were later merged into bigger ones. Every of these bigger cubes comprised 6 × 6 small cubes, so that 25 CT zones were obtained, as displayed in Fig. 1. Segmentation was verified by an experienced ophthalmologist, and in case of errors, they were manually corrected. Three-dimensional (3D) CT maps were created using Microsoft Excel (Microsoft Office 2019, Microsoft Corporation, Redmond, WA, USA). Left eyes were outcomes were transformed into right eye format.

Statistical analysis was performed using Statistical Package for Social Sciences (SPSS) software for Windows v.27 (IBM Corporation, Somers, NY, USA). Normality was checked with Kolmogorov–Smirnov test. Means and standard deviations were calculated for quantitative variables. ANOVA test was used for comparisons between groups, with Bonferroni *post-hoc* analysis. Bonferroni was used for multiple comparisons adjustment. Multiple lineal regression analyses were performed to analyze possible factor influencing CT. Statistical significance was established at *p* < 0.05.

## Results

60 eyes of 60 patients with SLE treated with HCQ, and 54 eyes of 54 healthy subjects were recruited. All patients with SLE were treated with HCQ exclusively. None of the patients received any other medications. Demographic data are displayed in Table 1. There were no differences in age, BCVA, IOP, SE or AL.

**Table 1:** Demographic data

	Study group		*p*
	Healthy	SLE + HCQ	
Female/male participants, number	43/11	59/1	
Right/left eyes, number	31/23	30/30	
Age, years	43.79 ± 8.98	45.16 ± 6.43	.346
BCVA, logMAR	− 0.03 ± 0.09	0.00 ± 0.12	.137
IOP, mmHg	14.72 ± 2.86	13.63 ± 2.76	.271
SE, D	− 0.82 ± 1.80	− 0.43 ± 1.33	.219
AL, mm	23.67 ± 0.87	23.52 ± 0.96	.400
SLE duration, months	–	125.58 ± 63.10 (120.05)	–
HCQ durantion, months	–	87.87 ± 52.13 (85.23)	–
Mean daily HCQ dose, mg	–	225.62 ± 85.71 (200.00)	–
Mean cumulative HCQ dose, g	–	605.05 ± 358.18 (586.70)	–

*SLE* systemic lupus erythematosus, *HCQ* hydroxychloroquine, *BCVA* best corrected visual acuity, *IOP* intraocular pressure, *SE* spherical equivalent, *AL* axial length

Outcomes are expressed as means ± standard deviations. Median values are displayed in brackets

Statistical *p* values are highlighted in bold

Table 2 shows CT in the 25 zones in both study groups with *p* values. We found differences in zones 9, 14, 15, 19 and 20, that is, in central-nasal locations. CT was higher in patients with SLE and HCQ than in healthy subjects. No segmentation errors were detected in any patient.

**Table 2:** Mean choroidal thickness with standard deviation in every choroidal zone

	Study group		*p*
	Healthy	SLE + HCQ	
Z1	249.38 ± 66.48	258.79 ± 67.39	.455
Z2	278.16 ± 62.74	289.02 ± 64.36	.364
Z3	284.17 ± 58.70	299.34 ± 67.52	.202
Z4	261.25 ± 65.57	280.41 ± 73.96	.145
Z5	226.36 ± 65.99	246.76 ± 77.20	.131
Z6	226.95 ± 68.19	233.30 ± 69.59	.624
Z7	264.34 ± 60.73	277.74 ± 64.75	.257
Z8	277.45 ± 60.08	295.47 ± 69.96	.142
Z9	251.89 ± 66.00	278.36 ± 73.51	**.045**
Z10	210.85 ± 75.93	232.90 ± 77.29	.128
Z11	218.83 ± 68.44	221.83 ± 67.45	.815
Z12	259.47 ± 62.40	268.14 ± 67.25	.477
Z13	273.06 ± 63.28	293.41 ± 69.28	.104
Z14	248.30 ± 70.58	282.84 ± 76.81	**.014**
Z15	205.36 ± 82.08	238.18 ± 84.50	**.038**
Z16	220.48 ± 74.58	215.17 ± 66.23	.690
Z17	249.74 ± 69.67	253.43 ± 69.02	.777
Z18	259.36 ± 68.86	276.34 ± 75.12	.213
Z19	236.68 ± 72.73	267.36 ± 78.73	**.033**
Z20	196.07 ± 79.93	232.06 ± 82.09	**.020**
Z21	211.93 ± 74.60	215.85 ± 67.05	.769
Z22	239.71 ± 72.27	245.83 ± 70.76	.649
Z23	254.86 ± 72.16	260.72 ± 77.44	.676
Z24	236.49 ± 69.84	250.41 ± 80.69	.326
Z25	204.83 ± 73.04	228.20 ± 82.72	.112

*SLE* systemic lupus erythematosus, *HCQ* hydroxychloroquine

Statistical *p* values are highlighted in bold

**Fig. 2:**
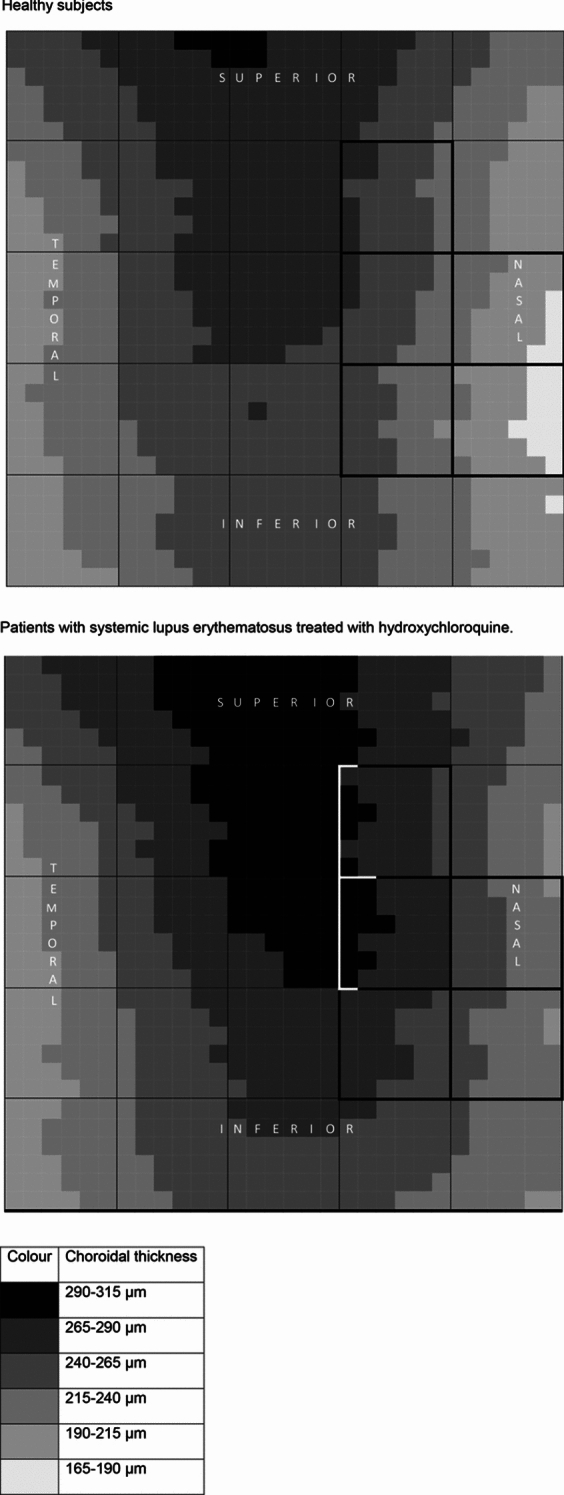
Two-dimension choroidal thickness representations on a right-eye model

Figure 2 shows CT in right eye format in healthy subjects and patients with SLE and HCQ. These figures were created according to CT values obtained in the 30 × 30 cubes grid, that is, according to 900 macular CT values. Choroidal zones with statistical differences are highlighted.

**Fig. 3:**
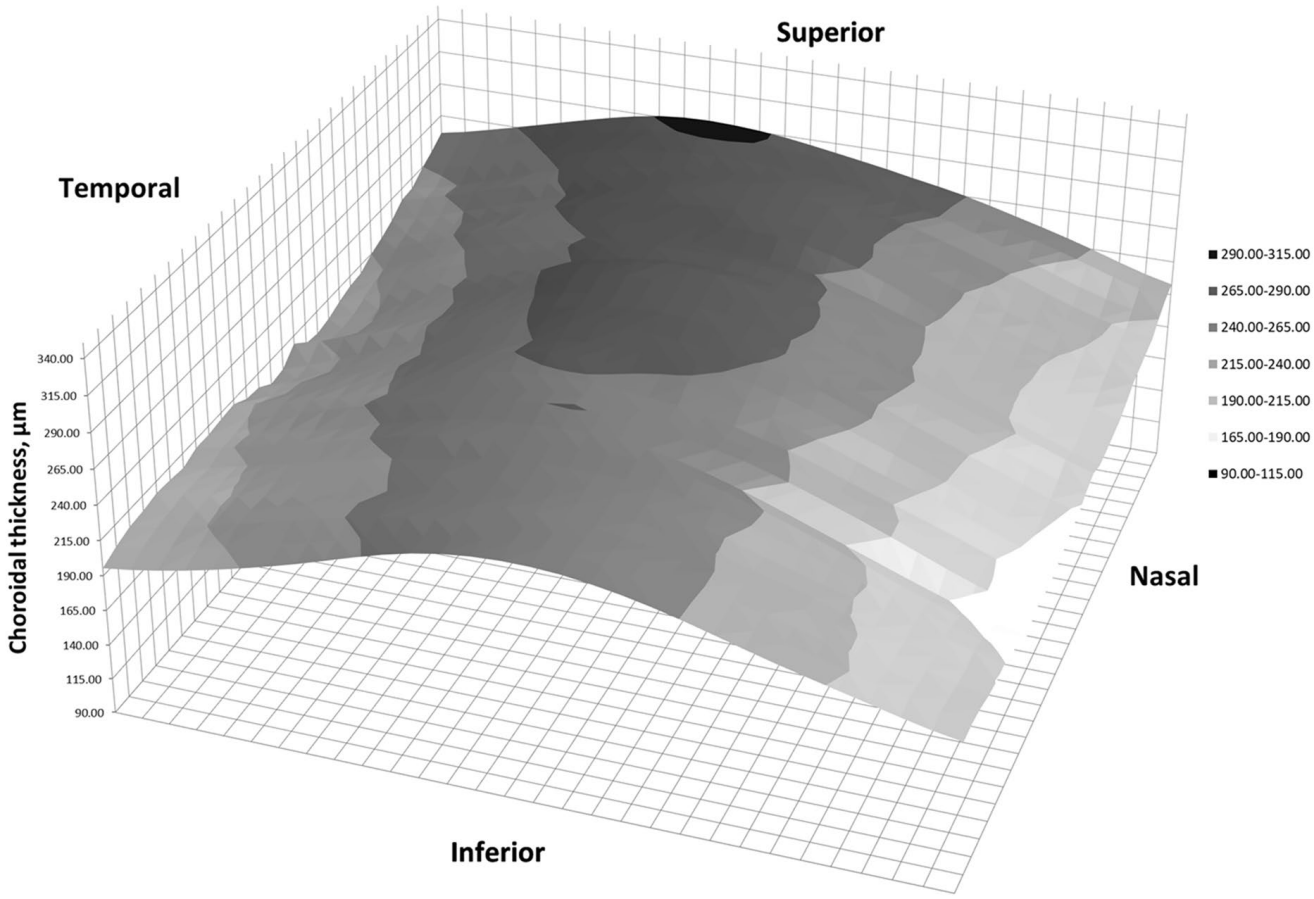
Two-dimension representation of choroidal thickness in a right eye of a healthy subject

**Fig. 4:**
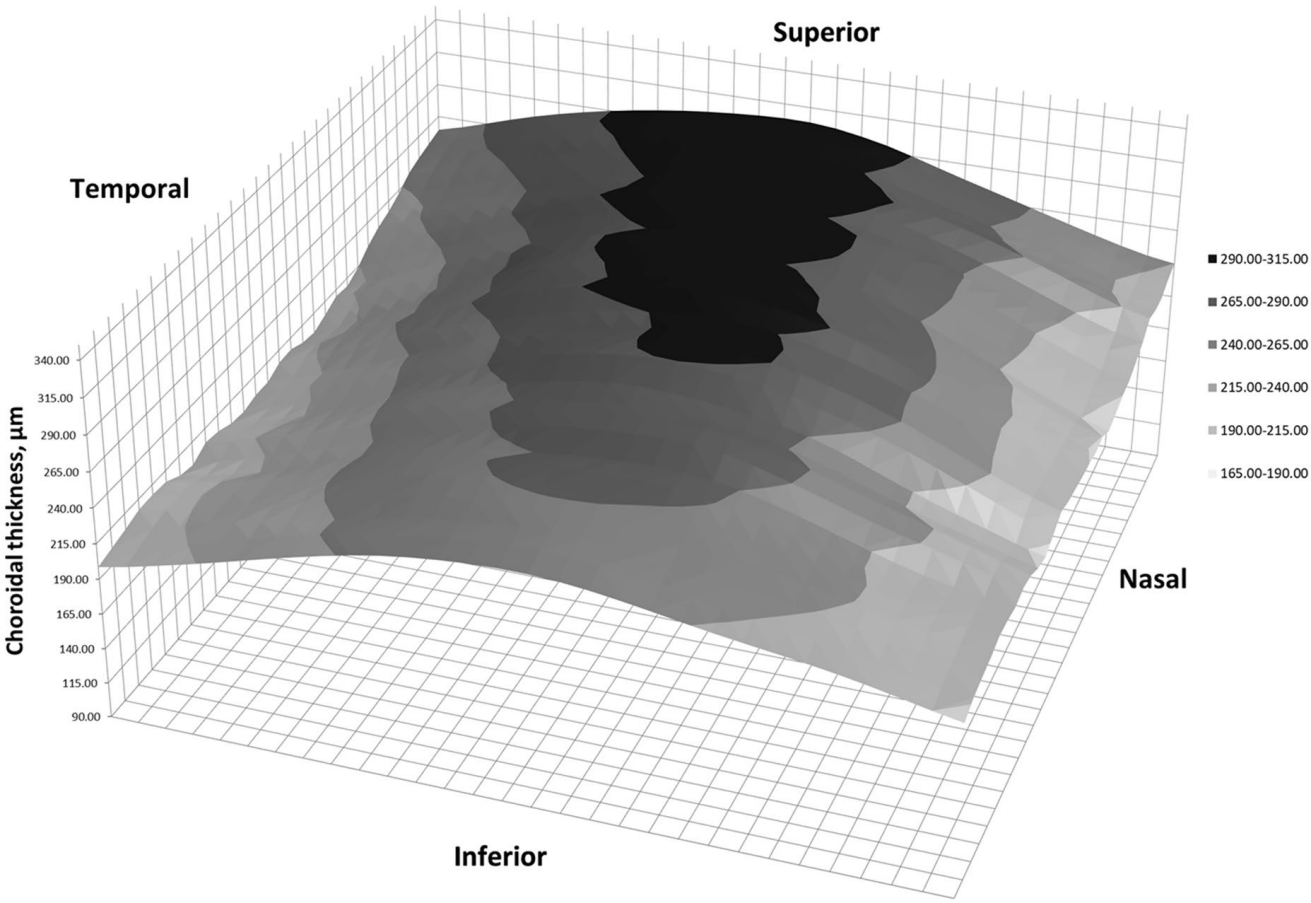
Three-dimension representation of choroidal thickness in a right eye of a patient with SLE treated with HCQ

Figure 3 shows three-dimensional (3D) representations of CT in healthy subjects, and Fig. 4 in SLE patients, in right eye format.

Multiple lineal regression analyses showed that neither SLE duration, nor HCQ duration, nor mean HCQ dose had any influence on CT in any of the 25 choroidal locations (*p* > 0.05). Cumulative dose of HCQ showed some relationship only with one of the 25 zones. Outcomes are displayed in Table 3.

**Table 3:** Outcomes of the multiple lineal regression analyses

Dependent variable	R^2^	Independent variable	β
Zone 6 (superior temporal)	0.08	Age	− 3.08 ± 1.42
Zone 10 (superior nasal)	0.09	AL	− 24.68 ± 10.28
Zone 13 (central)	0.08	AL	− 20.78 ± 9.31
Zone 14 (central)	0.12	AL	− 27.79 ± 10.11
Zone 15 (central nasal)	0.09	AL	− 26.89 ± 11.28
Zone 16 (central temporal)	0.11	AL	− 22.92 ± 8.74
Zone 17 (central)	0.15	AL	− 28.34 ± 8.92
Zone 18 (central)	0.19	AL	− 34.72 ± 9.42
Zone 19 (nasal)	0.27	AL	− 42.04 ± 9.66
		Cumulative dose of HCQ	− 0.05 ± 0.03
Zone 20 (nasal)	0.16	AL	− 34.65 ± 10.49
Zone 21 (inferior temporal)	0.08	AL	− 20.35 ± 9.01
Zone 22 (inferior)	0.09	AL	− 22.63 ± 9.45
Zone 23 (inferior)	0.12	AL	− 28.44 ± 10.16
Zone 24 (inferior)	0.17	AL	− 25.76 ± 10.43
		Age	− 3.21 ± 1.59
Zone 25 (inferior nasal)	0.10	AL	− 27.47 ± 10.92

*AL* axial length

## Discussion

Hydroxychloroquine is usually a first-line treatment for SLE [2, 3], but they have opposite effects on CT, even prior to ophthalmological affection. SLE seems to stimulate a thinner choroid [13–15], whereas HCQ seems to promote its thickening [9]. As far as we know, this is the first detailed study of the choroid in patients with SLE treated with HCQ. In addition, researchers usually use the ETDRS grid, but its area is smaller than the 6 × 6 mm square that can be analyzed when using the methods we described.

As expected, most of the participants of this cross-sectional study were female, because SLE is more frequent in females. Both eyes were expored because previous studies suggest that CT in right and left eyes is symmetric [16]. Triton DRI OCT is a SS-OCT, so it allows a deep and reliable analysis of the choroid, and its repeatability and reproducibility have been previously proven [17]. Although manual measurements may be adequate, automatic measurements usually show higher rates of reproducibility [18].

Choroidal thickness distribution in healthy controls was similar to previous studies, in which higher values have been reported in central and superior macula [10, 19]. An increase in CT was observed in nasal zones in patients with SLE and HCQ for at least one year. Those regions are exactly where the choroid is thinner in healthy subjects. Although the choroid is usually thicker in central and superior regions, no differences were found. Statistical analysis could not determine whether the variations on CT was due to HCQ or SLE. Due to ethical considerations, we could not enroll a third study group of patients with SLE without treatment, or even a fourth study group of healthy subjects under treatment with HCQ.

It is reasonable that patients with SLE enrolled in previous studies evaluating CT changes were under treatment with HCQ or another drug, but it is not clarified in most of them. This could explain why some authors found a thinning [7, 8], while some others found a thickening [13–15]. In case of lupus nephritis, a choroidal thickening takes place, despite not been correlated to the duration of the disease [15]. There is even a previous research whose authors could not detect any variation in CT in SLE patients withouth ophthalmological affection [20]. This could be explained after the small sample size of patients enrolled (20 SLE patients and 20 controls).

On the other hand, HCQ has been associated with thinner CT when being used for different conditions [9, 21], even prior to the development of HCQ retinopathy. In case of HCQ retinopathy, CT gets significantly thinned [21]. Nonetheless, it seems that CT changes can differ depending on the autoimmune disease, that is, some autoimmune diseases may cause a thinning, and some others a thickening in CT [14].

Other authors observed an increase in CT in patients with rheumatoid arthritis, and they explained this increase as a consequence of HCQ [9]. Some other studies evaluated CT variations in patients with SLE under different treatments, but they could not observe an association between HCQ and CT in those receiving HCQ [7]. In contrast with our study, most of these studies analyzed CT using either manual measurements on lineal slabs, or the ETDRS grid obtained with spectral-domain OCT. They did not provide any 3D representation of CT either. Thus, we cannot clearly distinguish whether the variations on CT are due to SLE itself or to its treatment with HCQ.

It should be remarked that increased CT is associated with active phases of SLE, as well as other autoimmune diseases [22]. None of the patients in our study had suffered from an active phase of SLE in the last year, but this might not have been considered in previous studies, and that might be a reason why opposite findings have been found regarding CT.

Arias-Peso et al. showed that the choroid was thicker in patients with SLE and HCQ for less than 5 years of treatment compared with healthy controls in some ETDRS sectors [12]. The main difference with our study is that they included patients with less than one year of treatment. In our study we discarded those patients to verify that HCQ had enough time to show some effects on the choroid. Additionally, they only analyzed ETDRS sectors, so parts of the choroid could not be evaluated.

Secondarily, study demonstrated the importance of analyzing the whole choroid in detail. Some parts of the choroid may not be evaluated when using the ETDRS grid. Finally, 3D representations help us detect all these changes in CT at a glance. This is the first time they are provided in patients with SLE or HCQ.

Three-dimension maps have already been published in other ophthalmological disorders, but never in case of SLE or HCQ. Multiple CT maps showed that CT has a different pattern than retina [10, 11, 23]. They also helped understanding CT in patients with high myopia [24], and demonstred local thickening in case of reticular pseudodrusen [25]. Additionally, they may have a role in diagnosing acute central serous corioretinopathy [26].

This study could not associate CT to duration of SLE, nor with duration of HCQ, nor with HCQ dose. Bayuk et al. performed a study comparing CT between SLE patients and healthy volunteers, and nor could they find any association between CT and disease duration, disease activity score, or HCQ dose [27]. Similarly, Braga et al. could not associate variations in CT in patients with lupus nephritis and duration of the disease [15].

Strengths of this study are the sample size and the detailed analysis of the choroid using automatic measurements. Patients older than 55 years were discarded, so cataracts did not interfere with our outcomes. Main limitations are that we could not study the effect of SLE and HCQ separately because HCQ is usually a first-line treatment for SLE [2, 3]. Further research should be performed to confirm our outcomes and it would be of interest to analyze the influence of SLE and that of HCQ on choroid separately.

In conclusion, patients with SLE treated with HCQ for one or more years present higher CT values than healthy subjects. Nasal zones seem to be the most sensitive to these changes, while the other choroidal locations remain similar. However, neither duration of SLE nor duration or dose of HCQ treatment seemed to have any influence on CT. Prospective longitudinal studies should confirm these outcomes.

## Data Availability

No datasets were generated or analysed during the current study.
